# COVID-19 vaccine equity in the Americas

**DOI:** 10.1016/j.lana.2022.100189

**Published:** 2022-01-21

**Authors:** The Lancet Regional Health– Americas

2 years have passed since the first COVID-19 case was reported. Since then, more than 5·5 million lives were lost due to COVID-19, and the world as we knew it has changed indefinitely. The development of vaccines by the end of 2020 marked the beginning of a slow recovery—while also exposing and exacerbating disparities in access to health care between the richest and poorest countries. As of Jan 12, 2022, 59·4% of the world's population have received at least one dose of a COVID-19 vaccine; however, 76·9% of those are from high-income countries while only 9·5% are from low-income countries. In this sense, the Americas are a perfect representation of the vaccine disparity in the world: while 86·6% of the Chilean population is fully vaccinated (ie, received all required doses for the vaccine scheme), only 1·08% of Haitians have received at least one dose of COVID-19 vaccine and fewer than 1% have completed the full vaccination scheme. Unequal vaccine and resource distribution resulted in a partially vaccinated world and failed to contain the pandemic. Now, as we start 2022, the world faces the threat of a new COVID-19 surge with the spread of the Omicron variant, and possibly more variants to emerge, while half of its population remains unprotected.

The slow and unequal deployment of vaccines not only exposed the already most vulnerable populations to a deadly infection, but also created islands of continuous high transmission that favoured the emergence of new variants. A month after being recognised as a variant of concern by WHO at the end of November, 2021, the newly-emerged Omicron variant has been identified in 110 countries across all six WHO regions. The new variant is driving a tsunami of COVID-19 cases, with the largest increase reported in the region of the Americas, where a 100% increase was observed for the week between Dec 27, 2021, and Jan 2, 2022, compared with the preceding week. By Jan 4, 2022, the USA registered a world record of 1 million new cases in a single day. Unique to Omicron is its high transmissibility and rapid growth. Preliminary studies suggest a significant degree of immune evasion and drop in vaccine effectiveness; however, vaccines continue to offer great protection against symptomatic disease, and Omicron cases have been associated with a reduced need for hospitalisation. Although the clinical severity of Omicron remains uncertain, the increased transmission imposes a potential for case surges that could overwhelm health-care systems, imposing a great burden to mentally and physically exhausted health-care workers and driving societies back to the brink of a collapse.

Omicron quickly became the dominant strain in North America and, in the past 3 weeks of December 2021, the state of New York (USA) registered a five-fold increase in hospitalisations of young children aged 5-11 years with COVID-19 symptoms—none of whom had been vaccinated. Additionally, as daily cases surge, those in high-risk categories will be affected the most. The spread of the Omicron variant can have a devastating impact in countries that are still falling behind in vaccination coverage such as Guatemala (36%), Jamaica (24%), Haiti (1·08%) and Suriname (44%). By the end of the first epidemiological week of 2022, the Omicron variant has been detected in at least 42 out of 56 countries and territories in the region and several of those countries – including Jamaica and Suriname - experienced a 100% or more relative increase in COVID-19 hospitalizations compared to the previous week.

Vaccines became available in the end of 2020, but the unequal distribution and variable levels of hesitancy jeopardised sufficient immunisation of the population. Currently, only 57% of the population in Latin America and the Caribbean is fully vaccinated, with many of received doses being donated by other countries through the COVAX mechanism. The emergence of a new variant with suggestive immune escape and potential for reinfection has triggered rich countries to endorse booster doses, attempting to increase their population's protection. But let us not forget how we got to this point: diverting the supplies from poorer, less vaccinated countries will only push us further away from ending the pandemic. As we get ready to face the third year of the pandemic, joint efforts should be directed to accelerating COVID-19 vaccination and expanding immunisation coverage globally, focusing on at-risk populations in all countries to achieve the WHO target goal to vaccinate 70% of the world by mid-2022. Leaders of governments and industry must work together to ensure consistent supply, deployment, and distribution of vaccines. For example, the lifting of intellectual property rights would enable local manufacturing supply, expand production capacity, reduce shipping costs, and expedite vaccination delivery. It is time to revisit, rectify, and refine our strategies, to approach the issue more efficiently and reach our goals. Unlike pills that are targeted to the individual, vaccines are a community strategy and only with timely and comprehensive mass immunisation of everyone eligible will the world be able to control viral transmission, protect those at increased risk, and start to envision the end of the pandemic.

